# Applications of leadless pacing

**DOI:** 10.1093/eurheartjsupp/suae089

**Published:** 2025-03-24

**Authors:** Larry Chinitz, Serge Boveda, Robin Richard-Vitton, Theofanie Mela

**Affiliations:** Cardiac Electrophysiology, NYU Langone Heart Rhythm Center, 530 1st Ave, HCC 6th floor, New York, NY 10016, USA; Heart Rhythm Management Department, Clinique Pasteur, Toulouse 31300, France; Brussels University VUB, Brussels, Belgium; Département de Rythmologie, Clinique Pasteur, Toulouse, France; The Demoulas Center for Cardiac Arrhythmias, Mass General Brigham, Harvard Medical School, Boston, MA 02114, USA

**Keywords:** Leadless pacemaker

## Abstract

Leadless pacemakers are being used with increased frequency due to improvements in the technologies, and the recognition of the substantial benefits over traditional transvenous devices. Current information shows a substantial reduction in morbidity with leadless devices which has resulted in a significant expansion in the indication for these devices. Patient selection now includes a younger population as well as those commonly excluded from consideration. Understanding these new applications will allow a larger group of patients to benefit from the significant advantages of leadless devices while not compromising quality and effective pacing. Further improvements in this technology will result in even more availability of these transformative devices.

The history of cardiac pacing begins in the first half of the 20th century with external pacemakers. Since that time, cardiac pacing has delivered substantial benefits to millions of patients suffering from bradyarrhythmia. The field has seen continuous advancements, particularly in the development of leads and batteries.^1^ However, leads remain the Achilles heel of pacing technology with short-term complications of transvenous pacing occurring in up to 12% of patients, primarily consisting of infections, pneumothorax, lead dislodgement, and haematoma. Long-term complications may affect up to 9% of patients, including lead failure, infection, venous obstruction, and tricuspid regurgitation (TR).^2,3^ Given that ∼1 million pacemakers are implanted worldwide each year, these complication rates translate into substantial morbidity. Leadless technology was developed to address these issues with the hope of significantly reducing the complication rates and providing therapy to patients either unable to receive or at high risk for complications with transvenous devices.

Leadless pacemakers were first proposed in the 1970s^4^ and have gained increasing prominence in clinical practice among electrophysiologists over the past decade. Landmark and pivotal trials on leadless pacing are summarized in *Table 1*. The longest-standing leadless pacemaker (LP) on the market, the Medtronic Micra VR, was launched in 2015. Medtronic then introduced the Micra AV in 2020.

**Table 1:** Leadless device trials

Study	Device	Innovation	Patients number	Mean follow-up (months)	Implantation rate success (%)	Complication rate (%)	Pericardial effusion (%)	Device embolization (%)	Elevated threshold needing reintervention (%)	Other	Refs no.
LEADLESS Reddy *et al.*	Nanostim	1st leadless	33	3	97	6	3	0	0	—	^5^
LEADLESS II Reddy *et al.*	Nanostim		526	6	95.8	6.5	1.5	1.1	0.8	—	^6^
Micra IDE Reynolds *et al.*	Micra		725	6	99.2	4.0	1.6	0	0.3	Control group of TV PM, complication rate HR 0.49	^8^
Micra PAR El-Chami *et al.*	Micra		1809	51.1 months	99.1	4.5	0.4	0.06	1.4	Switching implantation site from apex to septum	^9^
MARVEL 2 Steinwend *et al.*	Micra AV	AV synchronization through atrial systole sensing	75	—	—	—	—	—	—	Median AV synchrony 94.3%	^12^
LEADLESS II IDE Reddy *et al.*	Aveir		210	14	98	6.8	1. 9	1.0	0	—	^15^
Aveir DR i2i Cantillon *et al.*	Aveir DR	AV synchronization using an atrial device	300	3	98.3	8.7	0.7	3.4	0.3	Mean AV synchrony time 95%	^16^
SOLVE CRT Singh *et al.*	WiSE	CRT with an LV leadless and a TV RV lead	183	6	—	19.1	3.8	3.8	0	6.6% of pocket related complication	^20^
MODULAR ATP Knops *et al.*	EMPOWER	ATP delivery in communication with a S-ICD	293	6 (for 162 patients)	100	9.9	1.2	0	0	31 ATP delivery with tachycardia termination in 61.3%	^21^

AV, atrioventricular; TV, transvenous; PM, pacemaker; HR, hazard ratio; CRT, cardiac resynchronization therapy; LV, left ventricle; RV, right ventricle; ATP, anti-tachycardia pacing; S-ICD, subcutaneous implantable cardiac defibrillator.

The leadless Abbott AVEIR VR system came onto the market in 2022. Even more recently, we have seen the arrival on the market of the Abbott AVEIR DR dual-chamber leadless system.

Finally, it should be mentioned that there are two systems that are not yet available on the market but which are being used in clinical studies: the WISE CRT produced by EBR to stimulate the left ventricle (LV) endocardially, and the EMPOWER, produced by Boston Scientific, coupled with the EMBLEM S-ICD.

One of the main areas of improvement *with leadless devices* is the reduction of infectious risk. For transvenous pacemakers (TVPs), the infection rate has been estimated at 4.82 per 1000 device-years after primary implantation and 12.12 per 1000 device-years after device replacement.^5^ This type of infection is associated with a high risk of mortality; nearly one-third of patients who do not undergo extraction in the 1st month after diagnosis will die, and 18.6% of those who have an early extraction still face a risk of mortality, according to a Medicare registry.^6^ In contrast, no infections have been reported in any clinical trials of leadless pacemakers, and only limited individual cases of infection have been reported with the Micra device.^7^

Another area of improvement is lead or device malfunction. The Micra Coverage with Evidence Development (CED) study, a registry using Medicare data, compared the follow-up of patients with Micra and TVPs. At 3 years, the registry found that 1.9% of Micra devices experienced a breakdown or dislodgement, compared to 3.7% for TVPs.^8^

The most recent trials demonstrate a substantial reduction in the complication rates by as much as 63%. In a real-world setting, the Micra Post-Approval Registry (PAR) demonstrated excellent outcomes. In a cohort of 1817 patients, the implant success rate was 99.1%, with a 12 month complication rate of 2.7%. Additionally, there were fewer occurrences of pericardial effusions, which was attributed to targeting the right ventricular septum instead of the apex, as had been done before in the Micra IDE and Nanostim trials.^10^

However, leadless pacemakers initially provided only single-chamber ventricular pacing, which did not maintain atrioventricular (AV) synchrony. To address this limitation, the Micra AV was developed to preserve AV synchrony through the mechanical sensing of atrial systole.^11^ The MARVEL 2 study,^12^ which evaluated this accelerometer-based AV synchrony algorithm, found that effective AV synchrony was achieved more than 70% of the time at rest in 95% of the patients, with a median AV synchrony of 94.3%. The Aveir LP can pair with a second, similar device placed in the right atrium, enabling reliable AV synchrony while allowing atrial pacing. Communication between the devices is achieved through subthreshold electrical signals conducted via blood and myocardial tissue.^13^ The Aveir DR i2i study tested these dual-chamber devices in 300 patients, achieving a successful implantation rate of 98.3% and meeting the safety endpoint in 90.3% of the cohort. The performance endpoint was met in 90.2% of patients, with a mean atrial pacing threshold of 0.82 V for 0.4 ms. Atrioventricular synchrony was effective in more than 70% of the time for 97.3% of patients, with a mean AV synchrony above 95%.^14^

As a result of the early data on safety and efficacy, the indications for LP implantation have greatly expanded over the years. Initially, the device was mainly offered to older patients with persistent or permanent atrial fibrillation (AF) who only required demand ventricular pacing. Concomitantly patients with AV block who would only use pacing infrequently were also targeted. Pacemaker-dependent patients had been initially excluded from the IDE trials and therefore operators were hesitant to use LPs in such patients. Leadless pacemakers were also advocated for patients with vascular access issues with the capability of both femoral and internal jugular implantation. The internal jugular approach became Food and Drug Administration approved for the implantation of Micra devices in 2023. With further technological advances and expanded experience with LP implantation, new patient populations have been found to be suitable candidates for LPs. The ability to offer solely ventricular pacing or atrial sensing with ventricular pacing, as well as AV pacing has added significant flexibility in providing patients with the most appropriate device for their needs.

The extractability question has also been addressed with successful extractions through intermediate dwelling times without complications.^15,16^ Data regarding battery duration have confirmed the manufacturer’s initial estimates. A recent study found that the battery life actually often exceeds these expectations, with most patients experiencing an estimated battery longevity of more than 13 years.^17^ As a more recent development, the Micra AV2 LP has a longer projected lifetime of 16 years and automatically customizes AV synchrony settings for each patient, reducing the need for manual programming by more than 50% compared to Micra AV.

## Unique population candidates for leadless pacemaker placement

Patients on haemodialysis can greatly benefit from LP as the infection rate is much lower than with TVP, and there is no interference with the dialysis catheters and arteriovenous fistulas. In an analysis of 201 dialysis patients who underwent a Micra implantation, there were no cases of device-related infection after 6 months of follow-up.^18^ In a similar way, immunocompromised patients, such as patients post-cardiac transplant^19^ can benefit from LPs.

Patients with congenital heart disease may not be candidates for TVPs due to vascular access limitations. Small series have shown that a LP can be a safe and durable option in these patients.^20^

Elderly patients over 85 years of age or those with venous occlusion due to multiple pre-existing leads, who are at high risk for extraction due to their frailty and the chronicity of the leads, may safely be given a LP in place of their transvenous pacing system if they develop lead malfunction.^21^

Patients with a transvenous device infection and/or bacteraemia may also be good candidates for LP, if they require immediate pacing post-extraction. Small series and data from real life use of Micra LP have shown that the implantation of a LP during or after the extraction procedure is a safe and efficacious approach with no recurrent infections requiring device removal.^22,23^ The LP may become the final device that the patients receive or a transvenous device may be implanted after the infection is treated, if cardiac resynchronization and/or an implantable cardioverter-defibrillator (ICD) are needed.

Cardioinhibitory vasovagal syncope may be an indication for a LP even in younger patients. Avoiding TVPs, which may be associated with higher risk for lead-related complications (lead fracture which is more common in younger patients and venous occlusion), is certainly beneficial and LPs may suffice in most cases by offering back-up ventricular pacing with hysteresis. The battery life may even exceed the usual prediction due to the low burden of pacing. A small series^24^ has shown the feasibility and efficacy of this approach. The availability of an LP, however, does not diminish the difficult decision of pacemaker implantation in a young patient.

The ‘ablate and pace’ strategy for AF with uncontrolled heart rate management, especially in patients with advanced age, may include the implantation of an LP. Since the first report in 2017,^25^ this has become a common approach. Implanting the Micra LP toward the mid to apical septum is advisable, as the ablation catheter should be at least 35 mm away from the device to avoid heating of its metallic elements.^26^

Patients with tricuspid replacement (percutaneous or surgical) should preferably avoid having leads crossing the new valve. Therefore, a LP may be indicated as a more suitable and stable pacemaker compared to a new coronary sinus lead. Similarly, patients who develop severe TR due to a transvenous ventricular lead, may be considered for a leadless device after the extraction of the responsible transvenous lead.^27^

Leadless pacemakers are increasingly being used for conduction abnormalities following transcatheter aortic valve replacement. In a recent comparison of 730 patients who received LPs and 9608 patients with TVPs, the LPs had a lower rate of in-hospital complications and mid-term device-related complications compared to TVPs.^28^

Patients with *sinus node dysfunction* who experience symptomatic bradycardia may be candidates for a LP, particularly when pacing is only required for the right atrium. The Aveir device has been successfully deployed for single-chamber right atrial pacing.

Leadless pacemakers are currently not commonly used in paediatric populations due to size limitations. However, as miniaturization and advancements in battery technology progress, there is potential for leadless pacemakers to be adapted for *paediatric patients* with congenital or acquired bradycardia.

Although leadless pacemakers are generally designed for long-term use, there is potential for these devices to be adapted for *temporary pacing* needs, such as in acute care settings after cardiac surgery or during recovery from an acute myocardial infarction.

Biventricular pacing is a well-established treatment for heart failure in patients with a wide QRS complex^29^ but this may be the most significant obstacle to the adoption of leadless pacemakers. While leadless cardiac resynchronization therapy (CRT) is not yet a widely available treatment, research and development are rapidly progressing. If successful, this innovation could offer a less invasive, safer alternative to traditional CRT for patients with heart failure, reducing the complications associated with leads while improving the quality of life for those requiring biventricular pacing. To address this challenge, the WiSE (EBR) LP was developed, consisting of a tiny passive electrode placed in the LV, a subcutaneous transducer, and a separate battery. The WiSE-CRT system, first designed to be triggered by transvenous pacing, has also more recently been used in combination with a Micra LP placed in the right ventricle (RV). A recent small European registry found significant improvements with this system, including a shortening of the QRS duration (from 204 ms before intervention to 137 ms after) and an improvement in left ventricular ejection fraction from 28 to 39%, although there was no improvement in New York Heart Association functional class.^30^

As previously reported, this system has also been more widely used in conjunction with a conventional transvenous lead placed in the RV as part of the prospective multicentre SOLVE-CRT trial. This trial demonstrated a 16.4% reduction in left ventricular end-systolic volume and an 80.9% rate of freedom from complications at 6 months.^31^

Additionally, another LP, the EMPOWER (Boston Scientific), has been developed for use in combination with the EMBLEM S-ICD, to deliver anti-tachycardia pacing (ATP). The MODULAR ATP trial included 293 patients with an indication for ICD implantation. At 6 months, 97.5% of patients were free from major complications, and communication between the two devices was successful 98.8% of the time. During the follow-up period, 61.3% of arrhythmia episodes were successfully terminated by ATP.^32^

Certainly, a major drawback of LPs is the higher rate of pericardial effusion as compared to transvenous devices. In the Micra and Nanostim IDE studies and AVEIR LEADLESS II study, the rate of pericardial effusion was 1.5%. In the Micra PAR, the rate of pericardial effusion was lower at 0.77%.^10,33^ Though the rate of perforation in the Micra CED study was <1%, it was double the rate of perforation observed with transvenous devices (0.4%). Although the perforation rate with LPs appears comparable to or slightly higher than the perforation rate with TV-PPM, a major concern is the severity of these perforations. Fourteen percent of patients with perforation with Micra required rescue open heart surgery; two out of three perforations encountered with AVEIR also required rescue sternotomy.^10,33^ In an analysis of the MAUDE database, 27% of perforation events required sternotomy.^34^ These challenges will likely be exacerbated with the wider adoption of multi-chamber leadless pacemakers. It remains possible and early studies suggest that with increasing operator experience the frequency and severity of pericardial effusions will diminish. In addition, further developments in delivery systems will undoubtedly lower morbidity. Managing multi-chamber LPs adds a layer of complexity and risk to the management of these devices. It should also be stated that the current Aveir system does not offer remote monitoring and the Micra has limited storage capability.

It is possible that rechargeable battery technology will be soon available and make life cycle management of LP less complex. Researchers have successfully tested a heartbeat-powered pacemaker in living pigs.^35^ This may be an important step towards developing battery-free implantable medical devices. The new ‘symbiotic pacemaker’ consists of three components: a wafer-sized generator attached to the heart that converts the organ’s mechanical energy into electrical energy; a power management unit that has a capacitor to store that energy; and the pacemaker itself, which stimulates and regulates the heart muscle. These results are of great interest, but further development is critical. Other muscles could be utilized as a source of energy simplifying implantation techniques. Biological pacemakers modify non-pacemaker myocytes to provide automaticity using gene therapy technologies or add pacemaker syncytia to the heart through adult or embryonic stem cell therapies.^36^ Biological pacemakers are in the early stages of development and important challenges remain.

As discussed earlier, the single most important limitation to the adoption of leadless technology is the ability to achieve CRT. At present, most patients who require a high percentage of ventricular pacing are offered some form of CRT. Studies have shown that high septal pacing with leadless devices may provide a reduction in the width of the QRS complex and reduce the incidence of pacing induced cardiomyopathy.^37^ This is possibly the most significant in patients with preserved ventricular function who require frequent ventricular pacing. Additional studies are required to investigate the use of leadless technology, high on the interventricular septum, for patients with an ejection fraction more than 40%. Studies are also currently being performed on novel leadless pacemakers implanted in the vicinity of the conduction system or membranous septum. Comparison to traditional resynchronization therapy may provide important additional indications for leadless devices.

Leadless pacemakers are an exciting and developing technology with expanding indications. Short- and intermediate-term studies have demonstrated good feasibility, safety, and efficacy with favourable complication profiles as compared to transvenous devices. Improvements in leadless systems with the ability to address lifecycle management, cardiac perforation, rates of AV synchrony, and CRT will challenge conventional paradigms and greatly expand indications and suitable patient populations.

## Data Availability

No new data were generated or analysed in support of this research.
